# PROXIMAL: a method for Prediction of Xenobiotic Metabolism

**DOI:** 10.1186/s12918-015-0241-4

**Published:** 2015-12-22

**Authors:** Mona Yousofshahi, Sara Manteiga, Charmian Wu, Kyongbum Lee, Soha Hassoun

**Affiliations:** Department of Computer Science, Tufts University, 161 College Ave., Medford, MA 02155 USA; Department of Chemical and Biological Engineering, Tufts University, Medford, MA USA

**Keywords:** Xenobiotic metabolism, Environmental chemical, Molecular substructure, Phase I and Phase II enzymes

## Abstract

**Background:**

Contamination of the environment with bioactive chemicals has emerged as a potential public health risk. These substances that may cause distress or disease in humans can be found in air, water and food supplies. An open question is whether these chemicals transform into potentially more active or toxic derivatives via xenobiotic metabolizing enzymes expressed in the body. We present a new prediction tool, which we call PROXIMAL (*Pr*ediction *o*f *X*enob*i*otic *M*et*a*bo*l*ism) for identifying possible transformation products of xenobiotic chemicals in the liver. Using reaction data from DrugBank and KEGG, PROXIMAL builds look-up tables that catalog the sites and types of structural modifications performed by Phase I and Phase II enzymes. Given a compound of interest, PROXIMAL searches for substructures that match the sites cataloged in the look-up tables, applies the corresponding modifications to generate a panel of possible transformation products, and ranks the products based on the activity and abundance of the enzymes involved.

**Results:**

PROXIMAL generates transformations that are specific for the chemical of interest by analyzing the chemical’s substructures. We evaluate the accuracy of PROXIMAL’s predictions through case studies on two environmental chemicals with suspected endocrine disrupting activity, bisphenol A (BPA) and 4-chlorobiphenyl (PCB3). Comparisons with published reports confirm 5 out of 7 and 17 out of 26 of the predicted derivatives for BPA and PCB3, respectively. We also compare biotransformation predictions generated by PROXIMAL with those generated by METEOR and Metaprint2D-react, two other prediction tools.

**Conclusions:**

PROXIMAL can predict transformations of chemicals that contain substructures recognizable by human liver enzymes. It also has the ability to rank the predicted metabolites based on the activity and abundance of enzymes involved in xenobiotic transformation.

**Electronic supplementary material:**

The online version of this article (doi:10.1186/s12918-015-0241-4) contains supplementary material, which is available to authorized users.

## Background

When ingested, drugs and other foreign chemicals can be transformed by xenobiotic metabolizing enzymes, which are expressed throughout the body, in particular the liver and intestine. In mammals, including humans, clearance of xenobiotic chemicals from the body involves two to three phases, with the first two phases carrying out key structural modifications. Typically, Phase I activates the chemical by introducing a reactive and polar functional group, whereas Phase II conjugates the activated chemical with a charged species, increasing the molecular weight, reducing reactivity, and improving the transport property. An additional Phase III step can follow the conjugation step to eliminate the conjugated chemical from the cell into the extracellular medium. The enzymes mediating these reactions have broad specificity, and thus are capable of generating a variety of metabolic products. Cytochrome P450 (CYP) enzymes play an especially important role in Phase I modification, which often involves oxidizing the substrate by introducing a hydroxyl group or oxygen atom. Depending on the substrate, a CYP reaction can produce a highly reactive derivative that can bind and modify other molecules in the cell, including macromolecules, and thus pose a cytotoxicity risk [1].

In some cases, the products of xenobiotic transformation can be biologically active, and interact with endogenous enzymes or regulator molecules to interfere with critical physiological processes. An example where this might be a concern is in the case of emerging contaminants. For example, diethylhexyl phthalate (DEHP) is a widely used plasticizer that can be hydrolyzed in the body to form monoethylhexyl phthalate (MEHP). In vitro experiments using molecular and cellular assays have shown that MEHP can selectively activate the nuclear receptor peroxisome proliferator-activated receptor-γ (PPAR-γ) to promote adipogenesis [2], and thus could contribute to the development of obesity. Hydroxylated derivatives of polychlorinated biphenyls (PCBs) can inhibit the sulfation of thyroid [3] and steroid hormones [4], and thereby disrupt an important mechanism for regulating the levels of these hormones. In the case of PCBs, hydroxylation can also enhance the toxicity of these chemicals [5], possibly through a mechanism involving oxidative DNA damage [6]. A similar increase in toxicity has also been reported to result from hydroxylation of polybrominated diphenyl ethers (PBDEs) [7].

One approach for identifying endogenously formed xenobiotic transformation products is to experimentally profile bodily fluids such as blood or urine for compounds that are structurally related to the xenobiotic chemical. For example, Dhakal et al. utilized mass spectrometry (MS) to identify several Phase I and Phase II derivatives of a PCB in urine and fecal samples from mice [8]. This metabolite profiling study utilized selective ion monitoring, a MS method for targeted analysis. While this targeted approach affords quantitative analysis, it may not be comprehensive. By definition, targeted analysis requires a priori knowledge of the chemicals of interest, and consequently is limited in its potential for discovery. Ideally, investigation of xenobiotic transformation is sufficiently comprehensive to characterize the breadth of metabolic products that can be derived from a chemical of interest, while also focused enough to robustly identify and quantitate the products. To this end, complementing experimental analysis with computational prediction of transformation products could be a powerful strategy to enhance the discovery potential of analytical experiments, as it has been suggested by several previous studies on experimental detection of biotransformation products [9–13].

Several computational approaches have been developed to predict xenobiotic transformations that result in structural modifications to the chemical. Examples of well-known approaches are UM-PPS [14–16], Meta [17–19] and Meteor [20–22]. The University of Minnesota Pathway Prediction System (UM-PPS) [14–16] is a rule-based method specifically developed to predict microbial catabolism of organic compounds. Based on curated information on microbial reactions cataloged in the University of Minnesota Biocatalysis/Biodegredation Database (UM-BBD) [23] and documented in the published literature, UM-PPS generates a set of rules that specify how predefined functional groups may be modified through a metabolic reaction. These rules are ranked based on their likelihood of occurring under aerobic biodegradation conditions and applied to the matching functional groups in the query compound to predict the compound’s metabolic products. Meta, another rule-based method, predicts xenobiotic transformations in mammals by generating rules compiled from reviews and textbooks [17, 19]. Given a query compound, Meta searches for a molecular fragment in the compound that is recognized by a specific enzyme and transforms it into a product fragment. Meta uses a genetic algorithm to optimize the rules based on experimental observations to improve the predictions [18]. For a given metabolite, Meteor predicts the possible transformation steps using a reasoning engine that has a knowledge base composed of generic rules [20, 21]. Several hundred (841) rules are derived from 217 known biotransformation reactions. The rules are assigned a rank according to the lipophilicity and molecular weight of the query metabolite [22]. Kirchmair et al. provide a comprehensive review of computational approaches for predicting outcomes of xenobiotic transformations [24]. A drawback of rule-based approaches is that they rely heavily on generic transformation rules, leading to a large number of predictions that may be difficult to evaluate and interpret. MetaPrint2D-react is another tool for predicting biotransformations [25]. MetaPrint2D-react is based on MetaPrint2D [25], which predicts site of xenobiotic metabolism using ‘circular fingerprints’ of reactant-product pairs. The circular fingerprint is determined by counting the number of each atom type at each depth or level (defined by covalent bonds) around the reaction center. Libraries are created based on the fingerprints, and each atom in the query molecule is searched against these libraries. Any atom with a match in the libraries is marked as a possible site of metabolism. MetaPrint2D-react augments the rules derived using the fingerprinting techniques with generic rules that are added manually. For example, the specific rule for acetylation (the acetyl group is most common acyl group to be added) is supplemented with a generic rule that captures other cases of acylation.

We present in this paper a new method, termed PROXIMAL, for predicting the biotransformation of xenobiotics by human enzymes. PROXIMAL analyzes Phase I and Phase II xenobiotic transformations that are cataloged in public databases (i.e. DrugBank [26–28] and KEGG [29, 30]), and builds look-up tables linking specific molecular substructures with matching biotransformation operations that modify these substructures. To achieve specificity, the look-up tables take into account molecular substructures that consist of a reaction center and its two-level nearest neighbors. Given a query compound, PROXIMAL applies a select set of transformations from the look-up tables at one or more matching sites, or reaction centers, of the query compound. PROXIMAL then ranks the transformation results based on the activity and abundance of the enzymes involved in the transformations. To evaluate the predictive power of PROXIMAL, we investigate two case studies involving bisphenol A (BPA) and 4-chlorobiphenyl (PCB3), two environmental chemicals with suspected endocrine disrupting activity.

## Methods

The PROXIMAL method has three steps. The first step catalogs known xenobiotic transformation reactions (enzymes, substrates and products) recorded in databases. In this study, we focused on CYP enzymes (Phase I enzymes) and transferases (Phase II enzymes), which account for the bulk of xenobiotic chemical modifications and conjugations in mammals. Specifically, CYP enzymes catalyze about 75 % of these reactions [31]. The cataloged information is used to build look-up tables that associate a particular molecular substructure with a specific pattern of modification or conjugation. The second step uses the look-up tables to apply a select set of transformations to a matching substructure within the chemical of interest. Depending on the chemical, this step may generate a large number of possible transformation products due to the number of sites available for modification. The third step ranks the predicted transformation products using available data on the activity and abundance of the enzymes associated with the transformations.

### Step 1: Creating look-up tables

The look-up tables are created in four steps. In step 1a, we mine reaction databases to identify a list of relevant CYP reactions. In step 1b, we analyze the structural similarities between the reactants and products of the CYP reactions. In step 1c, we extract one or more transformation pattern and its neighborhood from each CYP reaction. In step 1d, we store these transformations in lookup tables. We explain each of the steps in detail below.

In step 1a, the list of biotransformation reactions was generated by mining DrugBank [26–28] and KEGG [29, 30]. Most of these reactions are catalyzed by CYP oxidoreductases, which are representative of enzymes that carry out Phase I reactions. In addition to CYP enzymes, the list also included several transferases that play a major role in Phase II metabolism. These enzymes are: UDP-glucuronosyltransferase (UGT; EC 2.4.1.17), sulfotransferase (SULT; EC 2.8.2.1), N-acetyltransferase (NAT; EC 2.3.1.5), glutathione S-transferase (GST; EC 2.5.1.18), thiopurine S-methyl transferase (TPMT; EC 2.1.1.67) and catechol O-methyl transferase (COMT; EC 2.1.1.6) [32]. At the time of completion of this work, DrugBank held data on 409 Phase I enzymes and 70 Phase II enzymes. KEGG held data on 154 Phase I enzymes and 61 Phase II enzymes. Each reaction is specified using a reactant-product pair.

**Fig. 1:**
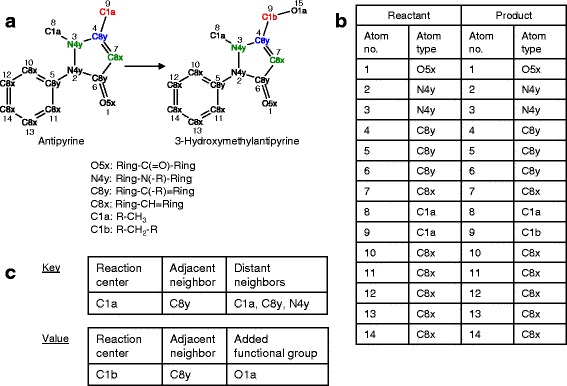
Schematic illustration of look-up table construction (PROXIMAL step 1). **a** A CYP reaction transforms the drug antipyrine to 3-hydroxymethylantipyrine, both represented in KEGG atom type format. Red, blue and green atoms are the reaction centers, adjacent neighbors and distant neighbors, respectively. The .mol files were downloaded from DrugBank. **b** List of matched atoms for the reactant and product. **c** The transformation look-up table has two parts, consisting of a key that specifies the modified reactant substructure and a corresponding value that describes the modifications resulting in the product

In step 1b, each reactant-product pair is examined, and patterns that express how reactants are transformed into products are identified. To analyze structural similarity between a reactant and product, we use SIMPCOMP [33]. SIMCOMP treats each molecule as a graph consisting of vertices (atoms) and edges (covalent bonds). Given a reactant and a product, SIMCOMP searches for the largest subgraph common to the two corresponding graphs, using heuristics to accelerate the search. The matched atoms in the common subgraph form the basis for aligning the reactant and product molecules. The output of SIMCMOP is a list of aligned atoms that comprise the subgraph. Figures 1a and b illustrate how SIMPCOMP analyzes the structural similarity between antipyrine and its CYP reaction product 3-hydroxymethylantipyrine. In Fig. 1a, each atom in the reactant and product is designated a KEGG atom type [34]. The atom type specifies the chemical element of the atom and its adjacent atoms connected by a covalent bond. For example, atom type C1a refers to a carbon atom connected to a functional group and three hydrogen atoms (R-CH_3_). Atom type C1b refers to a carbon atom connected to two functional groups and two hydrogen atoms (R-CH_2_-R). With antipyrine and 3-hydroxymethylantipyrine as inputs, SIMPCOMP returns as the output a list of atoms that align across the reactant and product (Fig. 1b). Each row in the output table holds an atom in the reactant and the corresponding atom in the product as determined by SIMCOMP based on the subgraph calculation. The ordering of the rows is determined by the atom order (numbering) in input file describing the reactant molecule. In the example of Fig. 1, all aligned atoms in the reactant and product have the same atom type, except atom number 9, which changes from atom type C1a in the reactant to C1b in the product.

In step 1c, the table of aligned atoms generated in the previous step is utilized to characterize the neighborhood of potential reaction centers. Each reactant atom that aligns with a product atom of a different atom type is a potential reaction center. The neighborhood of a reaction center consists of the reaction center atom’s adjacent and distant neighbors. An adjacent neighbor of an atom *x* is directly connected to atom *x* by a covalent bond. A distant neighbor of atom *x* is an adjacent neighbor of any one of atom *x*’s adjacent neighbors. From Fig. 1b, it can be seen that atom number 9 is a potential reaction center. This reaction center atom has only one adjacent neighbor, atom number 4, which is of atom type C8y. The distant neighbors are atom numbers 3, 7, and 9. The set of distant neighbors always includes the reaction center. Atoms 3 and 7 are type N4y and C8x, respectively. The reaction center (shown in red in Fig. 1a), its adjacent atom (shown in blue), and distant neighbors (shown in green) together comprise the substructure where the CYP-mediated chemical modification occurs.

In step 1d, information generated through steps 1a, 1b and 1c is entered into a lookup table, where information is stored as a key-value pair. The key consists of three fields: the atom type at the reaction center, atom types of adjacent neighbors, and atom types of distance neighbors. The value stores the corresponding transformation in three fields: the atom type of the product atom aligned with the reaction center, atom types of adjacent neighbors, and functional groups added to the product (atom types of any atoms adjacent to the reaction center in the product that are not present among the adjacent neighbors in the reactant). Figure 1c shows the key and value associated with the reaction center at atom number 9. Separate tables are created for Phase I reactions and Phase II reaction.

### Step 2: Generating potential products

Given a chemical of interest, PROXIMAL applies the transformation patterns represented in the look-up tables to generate possible products of Phase I and/or Phase II reactions. The chemical of interest is specified using an input .mol file. PROXIMAL processes this input file to represent the chemical in KEGG atom type format [34] using the KEGG API (Application Programming Interface). PROXIMAL treats all atoms in the chemical as possible reaction centers, and builds corresponding lists of adjacent and distant neighbors. A query is performed to identify any substructures that match to a key in the look-up tables. If there is a match, then the key’s value is applied to generate a biotransformation product. Depending on the number of matches, PROXIMAL may generate multiple products for a given chemical.

**Fig. 2:**
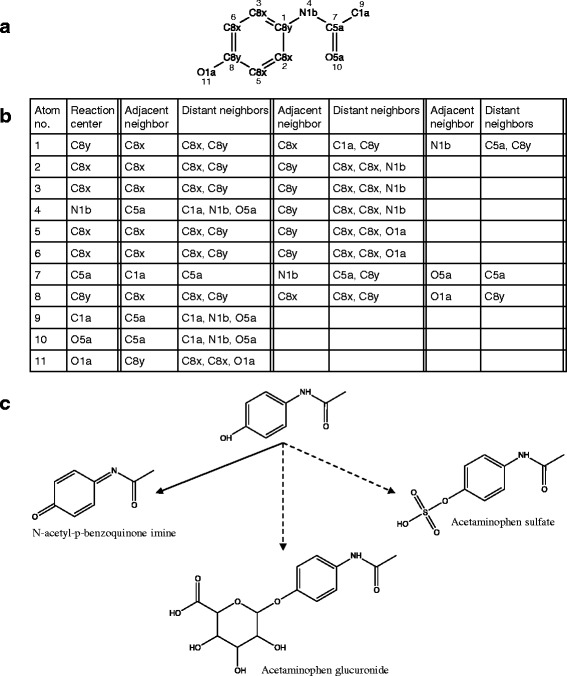
Schematic illustration of generating the transformation products (PROXIMAL step 2). **a** The example drug, acetaminophen, is shown represented in KEGG atom type format. The .mol file was downloaded from KEGG. **b** A list of atoms comprising acetaminophen and their adjacent and distant neighbors. **c** The products predicted to result from modifications of the reaction center at atom number 11 are N-acetyl-p-benzoquinone imine, acetaminophen glucuronide and acetaminophen sulfate

The use of the look-up table in predicting xenobiotic transformation products is illustrated through an example involving acetaminophen (Fig. 2). After converting the drug’s .mol file to the KEGG atom type format (Fig. 2a), PROXIMAL generates a list of potential reaction center atoms and their neighbors (Fig. 2b). Each row in the list is compared with keys in the Phase I and II look-up tables. In this example, we find matching keys for the 11th row (reaction center: O1a; adjacent neighbor: C8y; distant neighbors: C8x, C8x, O1a). Applying the corresponding value from the Phase I look-up table generates N-acetyl-p-benzoquinone imine. Applying the values from the Phase II look-up table generates two additional products, acetaminophen glucuronide and acetaminophen sulfate. All three predicted products have been experimentally confirmed in published studies [35, 36]. Figure 2c shows transformations pertaining to reaction center at atom number 11. Additional transformations are possible at other reaction center atoms, but are not shown in the figure.

### Step 3: Ranking the predicted metabolites

Several factors influence the likelihood that a particular transformation occurs. Two important, related factors are the enzyme’s (catalytic) activity and abundance [37]. Another factor is whether multiple enzymes can catalyze the same transformation. In ranking the predicted products, we assume that a transformation is more likely to occur if there are many different enzymes that can catalyze the reaction, and if the enzymes are highly abundant and active. Based on this assumption, we compute the following score for each predicted transformation product.

1 $$ score={\displaystyle {\sum}_k\left( average\kern0.5em  activity\right)\left( average\kern0.5em  abundance\right)} $$

Equation (1) sums the product of average activity and abundance for each enzyme that can catalyze the formation of the transformation product, with the relevant enzymes (index *k*) determined from the look-up tables. Values for average activity and abundance were obtained by analyzing published data. For this analysis, we focused on a subset of major Phase I (CYP) enzymes, as they play a quantitatively dominant role in human drug metabolism [38–40]. Specifically, we collected data on the following 9 CYP enzymes expressed in the human liver: 1A2, 2A6, 2B6, 2C8, 2C9, 2C19, 2D6, 2E1, and 3A4. Both abundance [38, 39, 41–43] and activity [44–48] data were obtained from multiple studies involving primary hepatocytes from human donors. The studies were carefully selected such that the substrates used to characterize activity were similar in structure (Additional file 1).

To afford quantitative comparisons of data taken from different studies, we accounted for missing values and normalized the data as follows. The activity and abundance datasets were each organized into a matrix, with rows corresponding to enzymes and columns corresponding to studies. All activity data were expressed in terms of pmol substrate converted/min/mg protein, and all abundance data were expressed in terms of pmol CYP/mg protein. In the case a study did not report the abundance or activity of an enzyme, the missing value was imputed based on the row average for the enzyme. As the imputed value affects the average, this value was later iteratively recalculated during our normalization routine until it converged within a reasonable tolerance (0.1 %). After estimating initial values for the missing data, each column in the activity or abundance matrix was scaled to vary from 0 to 1 by subtracting the minimum value from each column entry and dividing by the maximal range in the column.

2 $$ {a}_{new}=\frac{a_{{}_{ij}}-{a}_{j, \min }}{a_{j, \max }-{a}_{j, \min }} $$

In equation (2), *a*_*new*_ is the scaled activity or abundance, and the subscripts *i* and *j* refer to the enzyme and referenced study, respectively. Next, quantile normalization was performed on both abundance and activity matrices to normalize the distribution of data across studies. An iterative procedure was then applied using the results of the quantile normalization to recalculate the missing values initially estimated by averaging the values reported in the different studies. Once the values converged, a final value for CYP abundance or activity was calculated by taking a row average. These final, averaged values for each CYP were then normalized with respect to the sum of the final activity or abundance values for all 9 CYPs (Table 1).

**Table 1:** Abundance and activity values for CYP subfamilies after normalization

CYP	Abundance			Activity		
	Average	min	max	Average	min	max
1A2	0.0895	0.0089	0.1232	0.0414	0.0000	0.0773
2A6	0.1245	0.0906	0.1888	0.1393	0.1189	0.1664
2B6	0.0177	0.0000	0.0906	0.0814	0.0450	0.0976
2C8	0.1003	0.0906	0.1232	0.1441	0.0976	0.1664
2C9	0.2226	0.1888	0.2498	0.1198	0.0000	0.1541
2C19	0.0114	0.0000	0.0154	0.0580	0.0000	0.1541
2D6	0.0069	0.0000	0.0154	0.0985	0.0450	0.1664
2E1	0.1898	0.0994	0.2498	0.1669	0.1389	0.2018
3A4	0.2374	0.1888	0.2498	0.1506	0.0000	0.2018

Columns 2, 3 and 4 represent the normalized average, minimum and maximum CYP abundance values estimated from experimental data across multiple studies [38, 39, 41–43]. Columns 5, 6, and 7 represent the normalized average, minimum and maximum CYP activities estimated from experimental data across multiple studies [44–48]

## Results

To evaluate the effectiveness of PROXIMAL in predicting xenobiotic metabolism, we investigated two test cases involving the environmental chemicals bisphenol A (BPA) and 4- chlorobiphenyl (PCB3). BPA is a synthetic chemical that has been widely used as a plasticizer, and is present in numerous commercial and household products. The primary exposure route for humans is through ingestion of food and drink, as BPA leaches from plastic containers [49]. PCB3 is a persistent organic pollutant found in old electronic equipment, paints, plastics, glues and pesticides. Humans can be exposed to PCB3 through air, water or soil since the degradation rate in the environment is slow. Both chemicals can elicit biological effects in mammals that could pose potential health risks [3, 4, 6, 50]. However, it is an open question whether the observed effects are due to the parent chemical or the metabolic derivatives.

### BPA transformations

**Fig. 3:**
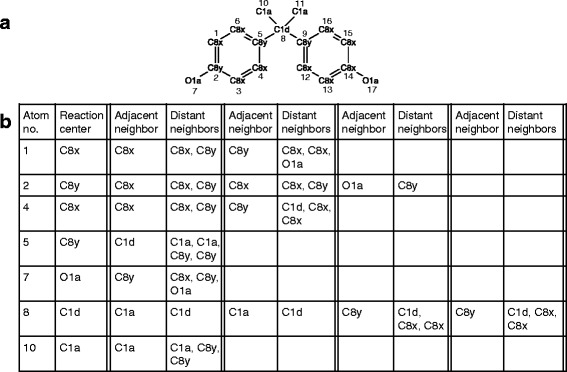
**a** Representation of BPA in KEGG atom type format. Each atom is represented by a number, which corresponds to the atom order in the .mol file (downloaded from KEGG), and its KEGG atom type. **b** Unique BPA atoms and their adjacent and distant neighbors are listed. Each atom of BPA is considered a reaction center, which can have up to four adjacent neighbors

**Fig. 4:**
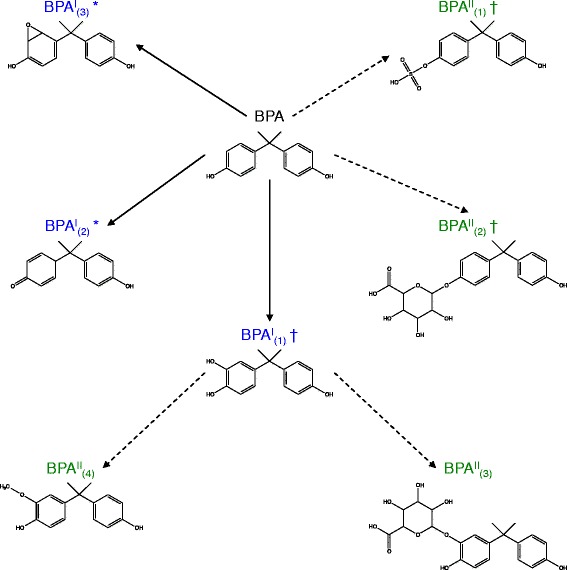
Predicted biotransformation products for BPA. The solid and dashed lines represent biotransformation through Phase I and Phase II, respectively. The symbol † indicates that the exact predicted compound has been verified by experimental data, whereas the symbol * indicates the predicted compound is similar to but not exactly the same as the structure reported in literature. The numeric subscripts identify the metabolites in the text

PROXIMAL identified a total of 17 molecular substructures in BPA (Fig. 3a). Due to the symmetries present in the molecule, only seven of these substructures are unique (Fig. 3b). All seven of these unique substructures have matching keys in the look-up tables generated using the Phase I and II reaction data from DrugBank and KEGG. Figure 4 shows only the predicted transformation products corresponding to the unique molecular structures that are unrelated to any others by symmetry. Five of the predicted derivatives (5-hydroxy BPA, BPA glucuronide, BPA sulfate, epoxide BPA, and bisphenol-o-quinone) have been experimentally verified in published reports.

Four of the seven predicted BPA derivatives result from modifications of the reaction center at atom 1 (first molecular substructure listed in Fig. 3b). This substructure consists of a reaction center in the aromatic ring (atom type C8x) and its neighbors (atom types C8x and C8y). Applying the matching key and value adds a hydroxyl group to the reaction center (Fig. 4, BPA^I^_1_). Studies by Schmidt et al. [51] and Jaeg et al. [52] detected the presence of 5-hydroxy BPA in liver microsomes and S9 fractions prepared from mice fed BPA. Applying matching Phase II transformations to the hydroxyl group reaction center resulting from the Phase I modification generates two additional derivatives (Fig. 4, BPA^II^_3_ and BPA^II^_4_). In addition to hydroxylation, another matching value adds an oxygen atom into the aromatic ring (Fig. 4, BPA^I^_3_). The resulting arene epoxide is essentially identical to a previously reported BPA derivative [51], except for the position of the epoxide group.

Another molecular substructure recognized as a key is the hydroxyl group (atom number 7) attached to the aromatic ring (5th molecular substructure listed in Fig. 3b). This key has one value in the Phase I look-up table. The modification specified by this value is to change the hydroxyl group into a carbonyl group (Fig. 4 BPA^I^_2_). The resulting derivative is similar to a previously detected quinol product [53], only differing by a missing hydroxyl group on the *ipso* carbon. Applying Phase II transformations directly on the hydroxyl group generates two conjugation products, BPA glucuronide and sulfate (Fig. 4, BPA^II^_1_ and BPA^II^_2_). Several studies have shown that BPA extensively metabolizes into BPA glucuronide and BPA sulfate in humans, rats, and mice [54–57].

After identifying the possible transformation products, we rank each predicted metabolite by computing a score that reflects the number of different enzymes that can carry out the predicted transformation as well as published data on the activity and abundance of these enzymes. As published data were more extensive for CYP enzymes compared to conjugation enzymes, we restricted the analysis to ranking only the Phase I products. The enzyme activity and abundance values used for this analysis are shown in Table 1. The min and max values represent the minimum and maximum of each row of the activity or abundance matrix obtained after the normalization iteration was completed. These values illustrate the possible range of normalized CYP activities/abundances, for each CYP, reported across different studies. The scores and rankings for the Phase I derivatives of BPA are shown in Table 2. The first and second columns of the table show the names of the derivatives using the nomenclature from Fig. 4 and the CYP enzyme families responsible for the transformation. We demonstrate the score calculation with an example. The transformation to 5-hydroxy BPA (BPA^I^_1_) can be catalyzed by any one of four CYP enzymes, namely 1A1, 1A2, 1B1, and 3A4. The average scores for the enzyme activity of 1A2 and 3A4 are 0.0414 and 0.1506, respectively (Table 1). Their corresponding enzyme abundance scores are 0.0895 and 0.2374. We calculated the score for BPA^I^_1_ as (0.0414_×_0.0895 + 0.1506_×_0.2374) = 0.0395. We did not include CYP 1A1 and 1B1 in the calculation since data for these enzymes were unavailable. The scores in Table 2 indicate that hydroxylated BPA is likely the dominant derivative with respect to the rate of formation via CYP enzymes. This ranking is consistent with a study by Schmidt et al. [51], which found that hydroxylated forms of BPA were the most abundant in liver microsomes. However, it is important to note that the abundance of a biotransformation product depends not only on its rate of formation, but also a number of other factors such as the product’s reactivity and availability of conjugation substrates involved in further biotransformation. Moreover, whether a metabolite is detected or not also depends on the sensitivity of the analytical method with respect to the chemical. In this light, the enzyme-based ranking should be interpreted as an estimate of one factor that influences the relative abundance of a biotransformation product.

**Table 2:** Score and ranking for the predicted products of Phase I biotransformation of BPA

Metabolites	CYPs	Score	Rank	Reference
BPA^I^_1_	1A1ˣ, 1A2, 1B1ˣ, 3A4	0.0395	1	[51, 52]
BPA^I^_2_	1A2, 2D6, 2E1	0.0361	2	[53]*
BPA^I^_3_	2E1	0.0317	3	[51]*

The first column shows the predicted compounds resulting from Phase I biotransformation. The notation is same as in Fig. 4. The second column indicates the CYP families responsible for the biotransformations. The superscript ˣ is added to enzymes for which activity/abundance data in human liver samples were unavailable, and the enzymes were thus not included in the score calculation. The 3rd and 4th columns show the calculated scores and rank. The last column lists the references reporting the predicted compound. The superscript * indicates that the predicted compound is similar to but not exactly the same as the reported structure

Nakamura et al. [53] reported observing hydroxycumyl alcohol (HCA) and isopropenylphenol (IPP) as the degradation products from BPA in both humans and mice. These two metabolites are produced through C-C bond scission via ipso-substitution [53]. However, PROXIMAL did not find HCA and IPP as derivatives of BPA. This is because PROXIMAL relies heavily on the transformations available in the database; therefore incompleteness of the databases directly affects its performance.

### PCB3 transformations

**Fig. 5:**
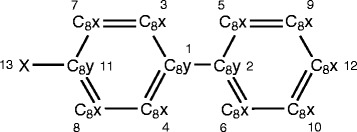
PCB3 representation in KEGG atom type format. Each atom is represented by a number, which corresponds to the atom order in the .mol file (from KEGG), and its KEGG atom type

**Fig. 6:**
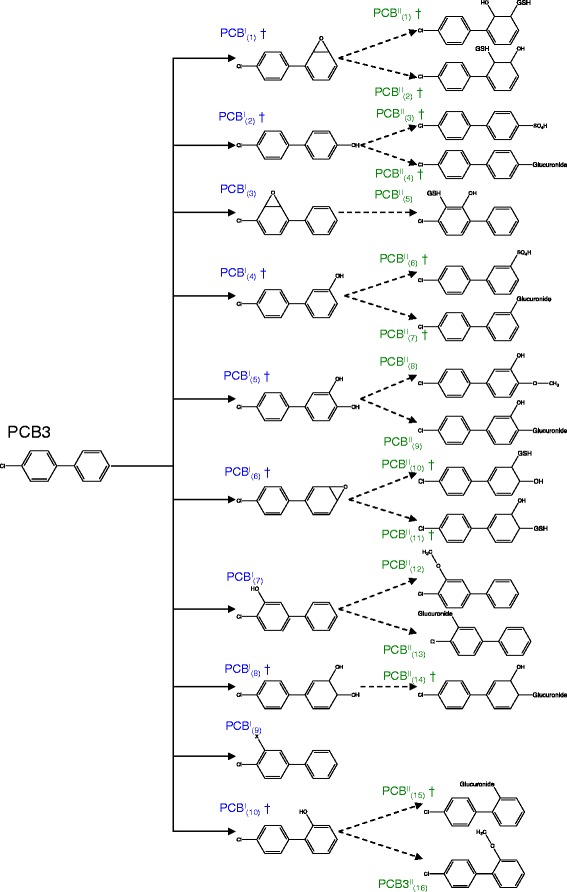
Predicted biotransformation products for PCB3. The solid and dashed lines represent the biotransformation reactions mediated by Phase I and Phase II enzymes, respectively. The symbol † indicates that the exact compound has been experimentally observed

The second case study analyzed the modification and conjugation of PCB3 via Phase I and II reactions. Figure 5 shows the atom numbers and atom types for PCB3. PROXIMAL identifies a total of 13 molecular substructures in PCB3 where only 8 of these substructures are unique. In total, PROXIMAL predicts 26 derivatives (Fig. 6).

Four of predicted PCB3 derivatives result from modifications of the substructure around atom number 12 (Fig. 5), which consists of a reaction center in the aromatic ring (atom type C8x) and its neighbors (atom types C8x and C8x). The derivatives are 4-hydroxy-PCB3 (Fig. 6, PCB^I^_2_), 3,4-dihydroxy-PCB3 (PCB^I^_5_), epoxide PCB3 (PCB^I^_6_) and cis-3,4-dihydro-3,4-dihydroxy-PCB3 (PCB^I^_8_). Each of these derivatives can be further transformed by applying matching conjugation steps identified from the Phase II look-up table. The hydroxyl group reaction center added via Phase I modification in 4-hydroxy-PCB3 (Fig. 6, PCB^I^_2_) generates 4-PCB3-sulfate (PCB^II^_3_) and 4-PCB3-glucuronide (PCB^II^_4_). Phase II derivatives of 3,4-diOH-PCB3 (PCB^I^_5_) include its glucuronated (PCB^II^_9_) and methylated conjugates (PCB^II^_8_). The epoxide group on the non-chlorinated aromatic ring of PCB3 (PCB^I^_6_) can be reduced and conjugated through Phase II enzymes to form PCB3 glutathione (Fig. 6, PCB^II^_10_ and PCB^II^_11_). Applying a Phase II transformation to cis-3,4-dihydro-3,4-dihydroxy-PCB3 (Fig. 6, PCB^I^_8_) generates a glucuronic acid conjugate.

Another major reaction center is one of the aromatic ring carbons (atom number 9). Applying Phase I modifications on this substructure generates an arene oxide PCB3 (PCB^I^_1_) and 3-hydroxy-PCB3 (Fig. 6 PCB^I^_4_). Like 4-hydroxy-PCB3 (PCB^I^_2_), 3-hydroxy-PCB3 can be further conjugated with a sulfate (PCB^II^_6_) or glucuronide group (PCB^II^_7_). Similarly, the arene oxide product (PCB^I^_1_) can also be conjugated with glutathione (PCB^II^_1_ and PCB^II^_2_) through Phase II enzymes.

The third Phase I reaction center is located at atom number 7. Modification of the corresponding substructure again produces a hydroxylated PCB3 (PCB^I^_7_) as well as 3,4-dichlorobiphenyl (PCB^I^_9_). Phase II transformation of the hydroxylated derivative can generate a methylated (PCB^II^_12_) or mono-glucuronide conjugate (PCB^II^_13_). The remaining Phase I transformation products, an epoxide (PCB^I^_3_) and 2-hydroxy-PCB3 (PCB^I^_10_), derive from modifications of reaction centers at atom numbers 3 and 5. The corresponding Phase II derivatives include a glutathione (PCB^II^_5_), glucuronide (PCB^II^_15_) and methylated conjugate (PCB^II^_16_).

In corroborating our predictions on PCB transformations with published reports, we expanded the literature search to include studies involving rodents, as there have been only few studies involving primary human liver cells. A recent study by Dhakal et al. [8] examined the metabolism and toxicity of PCB3 in male rats by analyzing urine samples collected following a bolus intra-peritoneal injection of the chemical. Using MS analysis, the authors identified several Phase I and Phase II products, including 2-, 3-, 4-hydroxy-PCB3, and their corresponding sulfate and glucuronide conjugates. With the exception of 2-PCB3-sulfate, these derivatives were also identified by our prediction method (Fig. 6). The same study [8] also reported the amount of 4-hydroxy-PCB3 (Fig. 6, PCB^I^_2_) in the urine samples was approximately 10 times greater than 3-hydroxy PCB3 (PCB^I^_4_). A separate study analyzing the distribution of hydroxylated PCB3 derivatives in rat liver microsomes found that the most abundant forms were, in decreasing order, 4-OH-PCB3, 3-OH-PCB3, and 2-OH-PCB3 [58]. This is in good agreement with the relative ranking of these three derivatives computed from enzyme activity and abundance data (Table 3).

**Table 3:** Score and ranking for the predicted products of Phase I biotransformation of PCB3

Metabolites	CYPs	Score	Rank	Literature
PCB^I^_1_	1A1ˣ, 1B1ˣ, 1A2, 2A6, 2B6, 2C9, 2C19, 2E1, 3A4	0.1172	1	[8, 58, 59]
PCB^I^_2_	1B1ˣ, 2B6, 2C8, 2C9, 2C18ˣ, 2C19, 2D6, 2E1, 3A4, 3A5ˣ	0.1113	2	[8, 58]
PCB^I^_3_	1A1ˣ, 1B1ˣ, 2C8, 2C9, 2E1, 3A4	0.1086	3	
PCB^I^_4_	2B6, 3A4	0.0372	4	[8, 58]
PCB^I^_5_	3A4	0.0358	5	[8]
PCB^I^_6_	2E1	0.0317	6	[8, 58, 59]
PCB^I^_7_	2E1	0.0317	7	
PCB^I^_8_	2C9, 2C19	0.0273	8	[8]
PCB^I^_9_	1A2	0.0037	9	
PCB^I^_10_	2D6	0.0007	10	[8, 58]

The first column shows the predicted compounds resulting from Phase I biotransformation. The notation is the same as in Fig. 5. The second column indicates the CYP families responsible for the biotransformations. The superscript ˣ is added to enzymes for which activity/abundance data in human liver samples were unavailable, and the enzymes were thus not included in the score calculation. The 3rd and 4th columns show the calculated scores and rank. The last column lists the references reporting the predicted compound

Additional derivatives predicted by PROXIMAL and experimentally confirmed in the study by Dhakal et al. include dihydrodiol (Fig. 6, PCB^I^8), dihydrodiol glucuronide (PCB^II^_14_) and 3,4-dihydroxy-PCB3 (PCB^I^_5_). Dhakal et al. also reported detecting molecules with mass signatures that correspond to the arene epoxide derivatives (Fig. 6, PCB^I^_1_ and PCB^I^_6_) and their glutathione conjugates (PCB^II^_1_, PCB^II^_2_, PCB^II^_10_ and PCB^II^_11_) predicted by PROXIMAL; however, these compounds could not be confirmed due to lack of pure chemical standards. Dhakal et al. [8] identified PCB3-mercapturic acid as a derivative product formed via degradation of PCB3-glutathione. While PROXIMAL correctly generated the glutathione conjugate (Fig. 6, PCB^II^_9_) as a transformation product, it was unable to predict further degradation of PCBII9 into PCB3-mercapturic acid, as KEGG or DrugBank did not include a CYP substrate with this type of reaction center.

In a separate study involving rat livers, Lehmann et al. [59] showed that PCB3 can be transformed into several active electrophiles, including arene oxides, which may bind to DNA, RNA and/or hemoglobin to cause cellular damage and increase the frequency of mutations. Transformation of PCB3 into arene oxide derivatives has also been observed in a study by McLean et al. [58] involving rat liver microsomes. Altogether, we were able to confirm 17 out of the 26 predicted PCB3 derivatives based on experimental data published in other studies.

### Comparing PROXIMAL with Other Tools for Metabolite Biotransformation

We provide two comparative studies of PROXIMAL with two tools, each presenting a different method for deriving biotransformation rules. The first tool, METEOR [20–22], utilizes a collection of knowledge-based biotransformation rules. The second tool, Metaprint2D-react [25], utilizes a data mining approach to characterize a specific atom environment followed by extensive manual curation of the data to organize the observed reactions into various categories (e.g., hydroxylation, expoxidation, methoxylation, etc.). Metaprint2D-react was selected for comparison, as it is most similar to PROXIMAL; although it differs from our method in that it requires manual curation to generate the transformation rules.

To compare PROXIMAL against METEOR, we applied PROXIMAL to the test cases reported in Button et al. [22]. The four test cases are Venlafaxine (antidepressant drug), Mianserin (antidepressant drug), Sulforidazine (antipsychotic drug) and Naltrexone (opioid antagonist). We compared the N-dealkylation products of these metabolites predicted by PROXIMAL, METEOR and reported in literature. Table 4 shows a summary of this comparison.

**Table 4:** Comparing PROXIMAL and METEOR

Test metabolite	PROXIMAL predictions	METEOR predictions [22]	Observed metabolites
Venlafaxine			[60]
Mianserine			[61]
Sulforidazine			[62]
Naltrexone			[63]

The first column shows the test metabolite. The second column shows predictions generated by PROXIMAL. The third column shows predictions generated by METEOR. The final column shows metabolites reported in the literature

As evident from the table, for the Venlafaxine case, PROXIMAL predicts two metabolites where one or both methyl group connected to the nitrogen is lost. Re-applying PROXIMAL on these derivatives also removes the methyl group from the ether group. Both of these derivatives are reported in mice and monkeys [60]. In contrast, METEOR only predicts the removal of one methyl group from the nitrogen atom. It does not predict removal of two methyl group from the nitrogen or removal of the methyl group from the ether group.

In the case of Mianserine, PROXIMAL predicts the formation of an N-desmethyl analog, similar to the result of METEOR. However, re-applying PROXIMAL to this derivative generates a product with an added hydroxyl group. This metabolite has been reported as a by-product of Mianserine in mice, rats, guine pigs, rabbits and humans [61].

In the case of Sulforidazine, PROXIMAL predicts the loss of an N-methyl group after applying human CYP enzymes to Sulforidazine. METEOR predicts the same metabolite. The removal of methyl group has been reported in literature as well [62], however, none of PROXIMAL and METEOR predicts the oxidation of sulfate which has been reported in rats [62].

For the last test case, naltrexonium, PROXIMAL predicts two metabolites, 7,8-dihydro-14-hydroxynormorphinone and 7,8-dihydro-14-hydroxynormorphine, generated by the cleavage of exocyclic nitrogen carbon bond and the loss of cyclopropylmethyl. Both of these metabolites are reported in literature [63] as derivatives of naltrexonium. METEOR predicts one of these metabolites.

To compare PROXIMAL with MetaPrint2D-react, we applied MetaPrint2D-react to the two cases BPA and PCB3. We utilized the suggested three different levels for fingerprint matching: Loose (matching exactly at 2 levels), Default (matching exactly at 3 levels), and Strict (matching exactly at 4 levels). In the case of BPA, MetaPrint2D-react generates 22 unique predictions when considering all levels, of which ten are uniquely generated at level 2, none uniquely generated at level 3, and three generated uniquely at level 4. PROXIMAL generates all level 4 predictions generated by MetaPrint2D-react, and two of the predictions that are generated using levels 2, 3, and 4. Metaprint2D-react does not predict BPA^II^_3_ in Fig. 4, which PRIXMAL predicts as the product of Phase II biotransformation. In the case of PCB3, MetaPrint2D-react generates over 100 predictions using the Loose prediction level, and over 32 predictions for the Strict prediction level. At level 2, MetaPrint2D-react identifies all derivatives predicted by PROXIMAL with the exception of PCB^II^_13_ and PCB^II^_15_ in Fig. 6.

## Discussion

In this work, we present a computational method, termed PROXIMAL, for predicting the transformation of xenobiotic chemicals by human CYP oxidoreductases and transferases. We evaluated the predictive power of the method by investigating case studies involving two prevalent environmental contaminants, BPA and PCB3, which are increasingly associated with developmental disorders and metabolic diseases. Overall, we found strong corroborating evidences in the literature for the predicted transformations of these two chemicals. In the case of BPA, we could confirm five of the seven predicted derivatives. In the case of PCB3, we confirmed 17 out of the 26 predictive derivatives, although we should note that the literature comparisons were based on studies that used animal models. Additional studies on PCB3 transformations in humans would be needed to further validate the predictions.

PROXIMAL uses information on the chemical neighborhood (atom types of two-level nearest neighbors) around the reaction center in constructing look-up tables as well as predicting the transformations. This confers a higher degree of specificity compared to generic rule based methods. On the other hand, this also limits the scope of products that can be predicted, as the predicted transformation will be similar to the reactions cataloged in the databases used to build the look-up tables. One potential consequence is that PROXIMAL could under-predict the true scope of products, resulting in more false negatives compared to a generic rule based method. For example, a comparison of PROXIMAL’s and MetaPrint2D’s outputs for BPA and PCB3 showed that PROXIMAL generates a significantly smaller number of predictions. Whether the use of specific rules leading to fewer predictions necessarily implies more false negatives is unclear, however, as rules that are too generic could over-predict the true scope of products and increase the risk of false positives. Of course, increasing the specificity does not abrogate the problem of false positives. Even with the specificity afforded by matching two-level nearest neighbors, PROXIMAL generated a large number of predictions for PCB3. For this reason, PROXIMAL includes an added ranking step that utilizes experimental (CYP abundance and activity) data drawn from outside of the databases used to build the look-up tables. The use of such orthogonal data is a distinctive feature, and differentiates this ranking method from other methods that rely on information contained in the databases used to develop the transformation rules (e.g., frequency of a particular transformation among a set of reactions within a database). The goal of the ranking is to estimate the likelihood of forming a particular predicted product relative to the other predicted products. Given a large number of possible products, it should be useful to guide the experimental validation by differentiating between predictions that are more or less likely to form via CYP reactions. Clearly, this ranking system cannot provide an estimate of the absolute probability a predicted product is indeed present in vivo, as this is influenced by other factors such as product reactivity, concentration of the source chemical, etc. Experimental data is necessary to train the ranking system and determine a threshold (cut-off) value that can be used as a reliable indicator of whether a predicted product will actually form at all. While PROXIMAL distinguishes itself from other tools in its level of specificity and in its ranking system, the comparison of PROXIMAL with other methods was based on a small set of experimentally verified test cases. The community would benefit tremendously from establishing a set of benchmarks that can be used to evaluate biotransformation prediction algorithms. Additionally, it is important to create a standard way of reporting the results.

It is important to point out that the lack of literature evidence does not necessarily imply that a prediction is false. It is possible that certain metabolic transformations of BPA and PCB3 have not yet been observed due to the instability of the products or some other difficulty in detecting these derivatives. Another reason could be that these products were outside the scope of a targeted analysis. One way to validate computational predictions is to perform untargeted metabolomics studies, for example using high-resolution MS. However, assigning a chemical identity to every ion detected in a full scan MS experiment remains a difficult task. In this regard, pairing experimental investigation of xenobiotic transformation with computational exploration would be extremely useful. Previously, several groups have utilized computational predictions of metabolite biotransformation to facilitate compound identification from MS data [10, 12, 13]. In these studies, the predicted chemical structures were used to calculate the expected mass signatures (accurate masses), which were then queried against experimentally obtained MS spectra. In addition to streamlining the data processing, computational predictions could also help avoid false negatives that result from relying on metabolite databases that may contain only a small, well-known subset of xenobiotic transformation products. For example, Ridder et al. [11] used computational predictions to generate an in silico library of biotransformation products resulting from human metabolism of polyphenols in tea, and found that only 23 % of the predicted products had entries in the Pubchem database. The predicted chemical structures can also be used to calculate isotope patterns and MS/MS fragmentation patterns, which are crucial in confirming the identity of detected ions, particularly when high-purity standards are unavailable for the chemicals of interest. For example, Pelander et al. utilized a prediction tool for biotransformation in conjunction with a prediction tool for ion fragmentation (ACD/MS Fragmenter) to discriminate between isobaric precursor ions of quetiapine products in human urine [10]. Finally, having a priori knowledge of the expected derivatives can guide the experimental workflow, including the choice of solvent for sample extraction and the method for chromatographic separation.

The present study focused on environmental pollutants to illustrate and evaluate our prediction method. In addition to organic pollutants, PROXIMAL could also be used for predicting transformations of other types of chemicals that contain substructures recognizable by Phase I and Phase II enzymes. Examples include drugs as well as various phytochemicals, e.g. phenolic compounds. Drug toxicity often arises from metabolic activation; i.e. the derivatives of a drug can be more toxic than the drug [64]. In this light, PROXIMAL could be used in conjunction with toxicity prediction software such as ADMET Predictor [65] and Derek Nexus [66] to assess the toxic potential of a drug compound’s possible derivatives that could form endogenously following the drug’s administration. Prospectively, this type of analysis could become part of an *in silico* screen designed to ultimately reduce the chance of drug-induced liver injury, which is a leading concern during drug development and testing [67, 68].

In addition to predicting the chemical structures of potential biotransformation products, PROXIMAL also provides a relative ranking of these products in terms of their likely occurrence. The ability to predict quantitatively dominant derivatives for a given chemical could complement experimental approaches, for example by informing the selection of metabolites for targeted analysis. For the two chemicals examined in our study, the predicted products with the highest ranking were also the derivatives that were frequently reported in the literature. This suggests that our ranking scheme could be biologically relevant. The caveat here is that the literature reports could reflect not only the prevalence of these derivatives, but also the level of interest in these compounds by the researchers. Clearly, a more thorough validation, for example using untargeted analysis, will be needed to evaluate the accuracy of the rankings.

An implicit assumption of our ranking scheme is that the likelihood of forming a particular derivative depends primarily on the kinetics of the biotransformation. We further assumed that the kinetics depends on the total abundance and activity of the enzymes involved. For example, we predicted that the hydroxylated forms of BPA are more likely to occur compared to other derivative forms. This result reflects the relatively large number (hence total abundance and activity) of CYP enzymes that can mediate the hydroxylation reaction. This prediction is consistent with experimental measurements on microsomes collected from pooled human liver samples [51].

Our ranking scheme does not take into account whether a particular transformation reaction is energetically more favorable, i.e. yields a more negative change in free energy, than other possible transformation reactions. Consequently, derivatives that are formed by the same set of enzymes will have the same rank. In contrast, derivatives formed by different sets of enzymes, including structural isomers, will have different rank. For example, our ranking scheme predicted that different hydroxyl isomers of PCB3 would be formed with different likelihoods. Specifically, we predicted that 4-OH-PCB3 is the most abundant derivative, followed by 3-OH-PCB3 and 2-OH-PCB3, in order. Interestingly, this prediction is consistent with experimental data [8, 58]. As shown in Table 3, CYP1B1 can catalyze the formation of 4-OH-PCB3 (PCB^I^_2_) but not the formation of 3-OH-PCB3 (PCB^I^_4_), whereas CYP3A4 can catalyze the formation of both isoforms. These differences between the two CYP enzymes reflect the reaction pattern information in the lookup tables, and imply that the extent of substrate flexibility varies from one CYP enzymes to another. It has been shown that CYP3A4 exhibits a large degree of flexibility, and can add a hydroxyl group to several different carbon atoms in a substrate molecule [69].

A limitation of our ranking method is that it does not include Phase II products. This was primarily due to insufficient information regarding Phase II enzymes. After an extensive literature search, we found only one study [48] that reported the specific activities of all six conjugation enzymes considered in the present work. As data become available, a ranking analysis could be performed based on relative enzyme activity and abundance similar to the analysis of Phase I enzymes. An additional factor to consider is that formation of the conjugation products depends on Phase I modification, as the atoms introduced in this step form the reaction centers for Phase II conjugation. As such, Phase I rankings may also need to be considered in ranking conjugation products. Yet another factor to consider is the availability of cofactors and conjugation substrates. For example, glutathione is a major antioxidant in the liver, and can become a limiting reactant under oxidative stress conditions.

Lastly, our ranking scheme did not include regulatory effects such as induction or inhibition of CYPs by the xenobiotic chemicals. It is well known that BPA can selectively induce or inhibit metabolic activity of certain CYPs. For example, Cannon et al. [14] reported on both inhibition and induction of BPA on CYP activity in human liver S9 fractions. One way to improve our ranking scheme is to include enzyme regulation to introduce an activity adjustment factor. For a given xenobiotic of interest, this factor would adjust the baseline CYP activities used in the present study to account for the inhibitory or inducing effects of the xenobiotic and its predicted derivatives. Clearly, this approach would require a substantial amount of additional information on the regulatory effects of xenobiotic chemicals. For the present study, we did not find sufficient data to confidently determine the inhibitory or inducing effects of BPA or PCB3 on the relevant CYP enzymes. As is the case for predicting the structural modifications resulting from biotransformation, a purely experimental approach will likely be intractable to determine the regulatory effects of xenobiotic chemicals on CYP enzymes, which may be mediated by ligand-activated nuclear receptors [70] controlling the expression of the enzymes. In this regard, computational approaches are warranted, for example to identify patterns in the chemical structures of known ligands for the regulatory molecules.

## Conclusions

We present in this paper a method to predict xenobiotic metabolism. To demonstrate the method, we applied the method to two case studies of endocrine disrupting environmental chemicals, and successfully predicted biotransformation products reported in the literature. We found experimental evidence for 71 and 65 % of the predicted BPA and PCB3 metabolites, respectively. Our method uses known chemical modifications found in reaction databases such as DrugBank and KEGG in conjunction with SIMCOMP to predict xenobiotic transformations for a given compound of interest. A novel aspect is the ability to rank the predicted metabolites based on available information regarding the activity and abundance of CYP enzymes. While the scope of this ranking was limited, we found good agreement between the predictions and findings reported in the published literature. In the discussion, we identify several limitations of the present prediction method that reflect the relative scarcity of information on Phase II enzymes and the regulation of xenobiotic metabolizing enzymes. Further studies, preferably in human cells, are warranted to further improve the predictive power and physiological relevance of PROXIMAL.

## Availability of supporting data

Supporting data were obtained from the Drug Bank database (http://www.drugbank.ca/), and the Kyoto Encyclopedia of Genes and Genomes (KEGG) database (http://www.genome.jp/kegg/). The source code for PROXIMAL will be made available upon request to the corresponding author.

## Additional file

Additional file 1:
**This file includes a list of substrates used in estimating cytochrome P450 enzyme activity.** The data is collected for the following 9 CYP enzymes: 1A2, 2A6, 2B6, 2C8, 2C9, 2C19, 2D6, 2E1, and 3A4. (PDF 120 kb)

## Abbreviations

PROXIMAL: Prediction of Xenobiotic Metabolism
CYP: cytochrome P450
PCBs: polychlorinated biphenyls
MS: mass spectrometry
BPA: bisphenol A
PCB3: 4-chlorobiphenyl
MEHP: monoethylhexyl phthalate
HCA: hydroxycumyl alcohol
IPP: isopropenylphenol
UM-PPS: The University of Minnesota Pathway Prediction System
