# Supplementary material for: PROXIMAL: a method for Prediction of Xenobiotic Metabolism
Source: BMC Syst Biol. 2015 Dec 22;9:94. doi: 10.1186/s12918-015-0241-4 (PMC4687097; doi:10.1186/s12918-015-0241-4)
Supplement: Additional file 1: — This file includes a list of substrates used in estimating cytochrome P450 enzyme activity. The data is collected for the following 9 CYP enzymes: 1A2, 2A6, 2B6, 2C8, 2C9, 2C19, 2D6, 2E1, and 3A4. (PDF 120 kb) [file 12918_2015_241_MOESM1_ESM.pdf]

Supplementary Table 1: Substrates included in estimating cytochrome P450 activity.

CYP	Substrate						
1A2	Phenacetin		Phenacetin	Phenacetin	Phenacetin, Resazurin	7-ethoxycoumarin	Caffeine
2A6			Coumarin	Coumarin	Coumarin	Coumarin	
2B6			Bupropion	Bupropion	Bupropion, Mephenytoin	7-ethoxycoumarin	
2C8			Amdiaquine	Amdiaquine	Paclitaxel	Paclitaxel	
2C9	Tolbutamide		Diclofenac	Diclofenac	Diclofenac	S-warfarin	Tolbutamide
2C19	S-mephenytoin		S-mephenytoin	S-mephenytoin	S-mephenytoin	S-mephenytoin	S-mephenytoin
2D6	Dextromethorphan		Dextromethorphan	Dextromethorphan	Dextromethorphan	Bufuralol	Bufuralol
2E1	Chlorozoxazone		Chlorozoxazone	Chlorozoxazone	Chlorozoxazone	7-ethoxycoumarin	Paranitrophenol
3A4	Midazolan	Testosterone	Testosterone, Midazolan	Testosterone, Midazolan	Testosterone, Midazolan	Testosterone	Testosterone
Referenced Study	[1]	[2]	[3] (200 donor pool)	[3] (50 donor pool)	[3] (16 donor pool)	[4]	[5]

References Cited

1. De Bock L, Boussery K, Colin P, De Smet J, T'Jollyn H, Van Bocxlaer J: **Development and validation of a fast and sensitive UPLC–MS/MS method for the quantification of six probe metabolites for the in vitro determination of cytochrome P450 activity**. *Talanta* 2012, **89**(0):209-216.
2. Ramachandran V, Kostrubsky VE, Komoroski BJ, Zhang S, Dorko K, Esplen JE, Strom SC, Venkataramanan R: **Troglitazone Increases Cytochrome P-450 3A Protein and Activity in Primary Cultures of Human Hepatocytes**. *Drug Metabolism and Disposition* 1999, **27**(10):1194-1199.
3. Shrivas K, Mindaye ST, Getie-Kebtie M, Alterman MA: **Mass spectrometry-based proteomic analysis of human liver cytochrome(s) P450**. *Toxicology and Applied Pharmacology* 2013, **267**(1):125-136.
4. Yamazaki H, Suzuki M, Tane K, Shimada N, Nakajima M, Yokoi T: **In vitro inhibitory effects of troglitazone and its metabolites on drug oxidation activities of human cytochrome P450 enzymes: comparison with pioglitazone and rosiglitazone**. *Xenobiotica* 2000, **30**(1):61-70.
5. Iyer KR, Sinz MW: **Characterization of Phase I and Phase II hepatic drug metabolism activities in a panel of human liver preparations**. *ChemBiolInteract* 1999, **118**(2):151.
